# ORBITA Trial: Redefining the Role of Intervention in the Treatment of Stable Coronary Disease?

**DOI:** 10.21470/1678-9741-2017-0243

**Published:** 2018

**Authors:** Luciano Cabral Albuquerque, Walter José Gomes

**Affiliations:** 1 São Lucas Hospital of the Pontifícia Universidade Católica do Rio Grande do Sul (PUCRS), Porto Alegre, RS, Brazil.; 2 Disciplina de Cirurgia Cardiovascular e Cardiologia da Escola Paulista de Medicina da Universidade de São Paulo (EPM-UNIFESP), São Paulo, SP, Brazil.

Although often overlooked, the primary goal for treating coronary artery disease is to decrease the risk of myocardial infarction (MI) and consequently reducing death, and the relief of anginal symptoms. While in the setting of acute coronary syndrome, the role of myocardial revascularization is well established, interventional procedures in patients with stable disease, especially percutaneous coronary intervention (PCI), remains controversial.

In this context, the recently published ORBITA trial^[^^1^^]^ in The Lancet journal, has brought important responses to this debate, involving unconventional and disruptive concepts in the field as placebo and nocebo effects. As the first blinded randomized, placebo-controlled trial to test the efficacy of drug eluting stents *versus* optimized medical treatment (OMT), the study enrolled 230 patients with stable angina and coronary artery stenosis ≥ 70% to receive a sham *versus* current PCI. During 6 weeks after the false or true procedure, both groups underwent to OMT, and the endpoints of exercise time, peak O2 consumption and control of angina were compared. Coronary lesions had mean area stenosis of 84.4%, and fractional flow reserve of 0.69. No significant difference in the primary endpoint of exercise time increment between groups (PCI minus placebo 16.6 s, 95% CI - 8.9 to 42 - *P*=0.2) was observed and also no deaths reported. Serious adverse events included four pressure-wire related complications in the placebo group, and five major bleeding events, including two in the PCI group and three in the placebo group. Contrary to the concepts derived from previous non-randomized studies, the authors concluded that in patients with medically treated angina and severe coronary stenosis, PCI did not increase exercise time by more than the effect of a placebo procedure.

These apparently surprising results are supported by an impressive methodological rigor and careful conduction of the study. The measurement of the placebo effect, with the creation of the sham group, is an innovation, and the researchers were impeccable in controlling bias. Another impressive point was the strict clinical control: for 6 weeks, patients could call a cardiologist 24 hour by 7 days a week, who would evaluate their medications and make sure they were understanding side effects. And medicines were adjusted a couple of times a week to get people maximally tolerated antianginal therapy.

As the key message, ORBITA delivered that the common clinical observation of symptomatic improvement from PCI might well contain a large placebo component.

Previously to the ORBITA trial, the benefits of routine revascularization in stable ischemic heart disease have been questioned, in terms of death and MI rates, in several randomized studies: Medicine, Angioplasty or Surgery Study (MASS II), Clinical Outcomes Utilizing Revascularization and Aggressive Drug Evaluation (COURAGE), Bypass Angioplasty Revascularization Investigation 2 Diabetes (BARI 2D) and more recently, the ongoing International Study of Comparative Health Effectiveness with Medical and Invasive Approaches (ISCHEMIA) trial.

In the MASS II trial^[^^2^^]^, 611 patients with proximal multivessel disease and documented ischemia were randomly assigned to coronary artery bypass grafting (CABG), PCI, or OMT. The 10-year mortality rates in the 3 groups were 25.1%, 24.9%, and 31.0%, respectively (*P*=0.09). The 10-year MI rates were 10.3%, 13.3%, and 20.7%, respectively (*P*<0.01). Freedom from angina at 10 years was 64% with CABG, 59% with PCI, and 43% with OMT (*P*<0.001).

In the COURAGE trial^[^^3^^]^, randomization of 2.287 patients to PCI plus OMT *versus* OMT alone did not reduce the long-term rate of death or MI. Nor, however, did PCI worsen prognosis, and crossover to PCI for progressive symptoms or acute coronary syndrome was required in 32% of OMT patients during a median 4.6-year follow-up. Moreover, patients randomized to PCI had less documented angina, were more likely to be angina-free (despite requiring fewer nitrates and calciumchannel blockers), and had improved quality of life for up to 3 years (8.35). The reduction in angina (assessed by the Seattle Angina Questionnaire) with PCI *versus* OMT was most evident only in those with the greatest level of baseline angina.

In the BARI 2D trial^[^^4^^]^, 2.368 patients with type 2 diabetes (90% with stable ischemic heart disease) were randomized to prompt revascularization with intensive medical therapy (MT) or to intensive MT alone, with randomization stratified by intended PCI *versus* CABG. The 5-year rates of death (the primary endpoint) and major adverse cardiac events (MACE - death, MI, or stroke) were not significantly different with either strategy. However, patients in the CABG stratum had more advanced coronary artery disease (including more triple-vessel and proximal left anterior descending coronary artery disease) than those in the PCI stratum, and patients randomized to CABG *versus* intensive MT had lower 5-year rates of MACE (22.4% *vs.* 30.5%; *P*=0.01), driven by less MI (7.4% *vs.* 14.6%). In patients with less extensive coronary artery disease, there was no difference in MACE with PCI *versus* intensive MT. Compared with intensive MT, prompt revascularization resulted in significantly greater freedom from angina for up to 4 years. Most measures of quality of life through the 4-year follow-up were also improved with routine revascularization compared with intensive MT only, and revascularization was ultimately required in 42% of MT patients during follow-up.

Of note, bare-metal stents (BMS) were used in most PCI *versus* MT trials, including the MASS II, COURAGE, and BARI 2D studies. In fact, first-generation drug-eluting stents (DES) may reduce recurrent ischemia compared with BMS, resulting in fewer hospitalizations for repeat revascularization. However, data from meta-analysis have been controversial to demonstrate advantage of DES in reducing late mortality or MI rate, comparing to BMS. Kirtane et al.^[^^5^^]^ evaluated the safety and efficacy of DES among "real-world" patients and those enrolled in pivotal randomized clinical trials (RCTs) and data from 9,470 patients in 22 RCTs and from 182.901 patients in 34 observational studies were included. In RCTs, no significant differences were observed in the long-term rates of death or MI after DES or BMS use for either off-label or on-label indications. Only in real-world observational studies, with greater numbers of patients but the admitted potential for selection bias and residual confounding, DES was associated with reduction of MI and mortality,

The specific evaluation of patients with stable coronary disease who underwent implantation of BMS or DES, even showed more intriguing results. In the Norwegian Coronary Stent Trial (NORSTENT) trial^[^^6^^]^, 9.013 patients were randomised to receive the implantation of DES or BMS. In the group receiving DES, 96% of the patients received either everolimus or zotarolimus-eluting stents. The primary outcome was a composite of death from any cause and nonfatal spontaneous MI, after a median of 5 years of follow-up. Secondary outcomes included repeat revascularization, stent thrombosis, and quality of life. There were no significant differences in the components of the primary outcome, and quality-of-life measures did not differ significantly between the two groups. Only the rates of repeat revascularization were lower in the group receiving DES.

On the other hand, the value of the decision making between aggressive or conservative approach of stable coronary disease, made after invasive study of coronary anatomy, has been questioned. In the ongoing International Study of Comparative Health Effectiveness with Medical and Invasive Approaches (ISCHEMIA) trial^[^^7^^]^ (NCT01471522), the main difference in study design is to randomize patients with stable coronary disease to invasive or conservative strategies, before catheterization. This is a multicenter randomized controlled trial with a target enrollment about 5.000 patients with at least moderate ischemia on stress imaging. The primary aim is to determine whether an initial invasive strategy of cardiac catheterization and optimal revascularization (with PCI or CABG, as determined by the local heart team) plus OMT will reduce the primary composite endpoint of cardiovascular death or nonfatal MI, in stable patients with moderate or severe ischemia and medically controllable or absent symptoms, comparing with an initial conservative strategy. Enrollment began in late 2012 and is projected to end in 2017. Average follow-up will be approximately 3 years. There are currently around 300 participating sites in more than 30 countries. The ISCHEMIA Trial thus aims to address limitations of previous strategy trials by: 1) enrolling patients before catheterization, so that anatomically high-risk patients are not excluded; 2) enrolling a higher-risk group with at least moderate ischemia; 3) minimizing crossovers; 4) using contemporary DES and physiologically guided decision making (FFR) to achieve complete ischemic (rather than anatomic) revascularization; and 5) being adequately powered to demonstrate whether routine revascularization reduces cardiovascular death or nonfatal MI in patients with stable ischemic heart disease (SIHD) and at least moderate ischemia. The results of the ISCHEMIA trial will have important implications regarding global guidelines for performance and reimbursement of revascularization procedures in patients with stable coronary disease.

However, while we wait the results of ISCHEMIA trial and other ongoing studies, the ORBITA study is the best designed trial comparing conservative and interventional strategies in patients with stable angina. The rigor with which the trial was carried out and the conclusion that PCI has a powerful placebo effect, should have impact in forthcomings guidelines of stable coronary disease.

## References

[1] Al-Lamee R, Thompson D, Dehbi HM, Sen S, Tang K, Davies J, ORBITA Investigators (2018). Percutaneous coronary intervention in stable angina (ORBITA): a
double-blind, randomised controlled trial. Lancet.

[2] Hueb W, Lopes N, Gersh BJ, Soares PR, Ribeiro EE, Pereira AC (2010). Ten-year follow-up survival of the Medicine, Angioplasty, or
Surgery Study (MASS II): a randomized controlled clinical trial of 3
therapeutic strategies for multivessel coronary artery
disease. Circulation.

[3] Weintraub WS, Spertus JA, Kolm P, Maron DJ, Zhang Z, Jurkovitz C (2008). For the COURAGE Trial Research Group. Effect of PCI on quality of
life in patients with stable coronary disease. N Engl J Med.

[4] Frye RL, August P, Brooks MM, Hardison RM, Kelsey SF, MacGregor JM, BARI 2D Study Group (2009). A randomized trial of therapies for type 2 diabetes and coronary
artery disease. N Engl J Med.

[5] Kirtane AJ, Gupta A, Iyengar S, Moses JW, Leon MB, Applegate R (2009). Safety and efficacy of drug-eluting and bare metal stents:
comprehensive meta-analysis of randomized trials and observational
studies. Circulation.

[6] Bonaa KH, Mannsverk J, Wiseth R, Aaberge L, Myreng Y, Nygård O, NORSTENT Investigators (2016). Drug-eluting or bare-metal stents for coronary artery
disease. N Engl J Med.

[7] International Study of Comparative Health Effectiveness with Medical and
Invasive Approaches (ISCHEMIA).

